# Effects of Combining Immune-Priming Sub-Lethal Low-Dose Radiation with 4-1BB Activation and Gal-3 Blockade in In Vitro and Preclinical Group-3 Medulloblastoma Models

**DOI:** 10.3390/cancers18121890

**Published:** 2026-06-10

**Authors:** Arabinda Das, Connor Stephenson, Daniel G. McDonald, Julian E. Bailes, David Cachia, Ramin Eskandari

**Affiliations:** 1 Department of Neurosurgery, Medical University of South Carolina, Charleston, SC 29425, USA; conner.stephenson@unchealth.unc.edu (C.S.); eskandar@musc.edu (R.E.); 2 Department of Radiation Oncology, Medical University of South Carolina, Charleston, SC 29425, USA; mcdonad@musc.edu; 3 Department of Neurosurgery, Endeavor Health, Evanston, IL 60201, USA; julian.bailes@endeavorhealth.org; 4 Pritzker School of Medicine, University of Chicago, Chicago, IL 60637, USA; 5 Department of Medicine/Hematology-Oncology, UMass Memorial Health, Worcester, MA 01605, USA; david.cachia@umassmemorial.org; 6 Department of Pediatric Neurosurgery, Medical University of South Carolina, Charleston, SC 29406, USA

**Keywords:** medulloblastoma, low-dose X-ray radiation (LDXR), Gal-3 inhibitors, 4-1BB activation, preclinical model

## Abstract

Background/Objectives: Pediatric group 3 (G3) medulloblastomas (MB) are therapy resistant and have a significantly worse prognosis than the other MB subtypes. Aggressive radiation/chemotherapy improves survival, but potential long-term comorbidities include neurocognitive deficits. In previous work, we demonstrated that low-dose X-ray radiation (LDXR) acts as an immunological adjuvant. Recent studies have demonstrated that galectin-3 (Gal-3) expression in MB tumors accelerates M2 macrophage infiltration and restricts T cell receptor (TCR)-mediated signaling. Immunotherapy with an agonistic anti-4-1BB monoclonal antibody (mAb) activates CD8+ T cells, promoting their survival and acquisition of potent cytolytic properties. Building on these findings, we hypothesized that immune priming via sublethal LDXR, combined with a Gal-3 inhibitor and an anti-4-1BB mAb, would boost anti-tumor effects, resulting in survival benefits. Methods: We tested this hypothesis in vitro in co-cultures of human MB cells and in vivo, in an immunocompetent G3MB mouse model (MP1). Treatment effects were assessed using Western blot, flow cytometry, hematoxylin and eosin (H&E) staining, immunofluorescence imaging, and analysis of cytokine and chemokine expression. Results: Our data demonstrated higher Gal-3 expression in MB patient-derived tumor tissue than in non-tumor tissue. LDXR modulated major histocompatibility complex molecules, and, combined with a Gal-3 inhibitor and an anti-4-1BB mAb, altered T-cell/tumor-cell interactions, enhanced T-cell-mediated MB cell death, and shifted cytokine production to drive microglial polarization toward the M1 subtype. Furthermore, H&E-stained tumor sections showed a ~70% reduction in tumor size compared with untreated controls. Conclusions: These preclinical findings suggest that combining immune priming with sublethal LDXR, Gal-3 inhibition, and 4-1BB activation may be an effective treatment strategy for G3MB.

## Simple Summary

Medulloblastoma is the most common malignant brain tumor in children. Group 3 medulloblastoma is the most difficult to treat and has the worst outcomes, partly because the tumor environment turns off immune responses. Our new idea for treatment was to use low-dose radiation to kick-start the immune system, then deliver two different antibodies directly to the tumor. One antibody blocks pathways that promote tumor growth, the other turns on immune pathways to attack tumor cells. We tested this combination therapy in cell cultures of different mouse brain cells and showed that it caused tumor cell death and activated anti-tumor immune cells but did not harm normal neurons. The treatment also caused immune cells to produce chemical signals against tumor growth. In mice with similar brain tumors, the treatment reduced tumor size and increased survival. This approach of combining therapies that work together may lead to new treatments for medulloblastoma.

## 1. Introduction

Medulloblastoma (MB) is the most common malignant brain tumor in children. It has historically been classified based on clinical markers (patient age, presence of metastases at diagnosis) and histopathological characteristics (classic, desmoplastic/nodular, or large cell/anaplastic) [1,2,3]. The Group 3 MB (G3MB) subgroup, representing about 25% of all MBs, is characterized by high MYC protein expression, resulting from somatic MYC gene amplification in 15–20% of cases. Despite extensive research, the successful treatment of G3MB remains a daunting challenge [1,2,3]. In addition, the high doses of radiation therapy (RT) given to all MB patients are associated with significant late morbidity [4,5]. Development of new, targeted therapies for G3MB must overcome several major obstacles: First, tumor-associated macrophages/microglia (TAMs) are an important determinant in the establishment of a T-cell-excluded tumor phenotype, limiting infiltration and invasion of the tumor by CD8^+^T cells [6,7,8]. Thus, TAMs play a significant role in creating an immune-protective tumor microenvironment (TME) that promotes tumor progression [6]. Second, it has been challenging to identify a specific therapeutic target present on G3MB cells but absent from normal cells, thereby placing surrounding neurons at risk of treatment effects. Third, G3MB tumors are heterogeneous; a targeted treatment may only kill a portion of the tumor cells [3]. To overcome these problems, new therapeutic approaches are needed.

Current cancer immunotherapies primarily aim to initiate or boost T-cell responses to tumor antigens. It is increasingly recognized that TAMs within the tumor are a critical determinant in the development of an immunosuppressive microenvironment, which allows tumors to evade the immune system [9,10]. Recently, studies have demonstrated the involvement of galectin-3 (Gal-3), a 29–35 kDa β-galactoside-binding protein, in cancer progression—augmenting tumor growth, invasiveness, metastatic potential, and immunosuppression [11,12,13,14]. Gal-3, produced by TME cells, also appears to be key to suppressing immune surveillance by killing T cells, increasing myeloid-derived suppressor cells (MDSCs) and CD4(+) foxp3(+) regulatory T-cells (Treg), polarizing TAMs from M1 to M2 phenotype, interfering with natural killer (NK) cell function, and supporting metastasis [11,12,13,14]. The overwhelming evidence of Gal-3 involvement in promoting tumor growth, metastasis, and immunosuppression makes Gal-3 an exciting, clinically relevant target for cancer immunotherapy [15].

Immunomodulatory monoclonal antibodies (mAbs) have demonstrated efficacy, either alone or in combination with other agents, in difficult-to-treat malignancies. These mAbs, directed against key molecular regulators on T cells or antigen-presenting cells (APCs), boost anti-cancer immunity by blocking inhibitory signals (checkpoint blockers) or by delivering therapeutic agents. It is well established that costimulatory receptor 4-1BB (CD137, TNFRSF9), a member of the tumor necrosis factor receptor superfamily, is expressed on activated immune cells, including CD4+ and CD8+ T cells. However, the ligand for 4-1BB activation is expressed predominantly by proinflammatory APCs, suggesting that 4-1BB stimulation sources are limited in the highly immunosuppressive TME. However, 4-1BB can be ligated by soluble agonistic anti-4-1BB mAb to promote antitumor immunity; such antibodies have been shown to eradicate established large tumors in vitro and in vivo [16,17,18].

Recent observations suggest that RT may expand the therapeutic reach of immunotherapy by increasing the tumor mutational burden, thereby priming the tumor for further immunotherapies and activating resident macrophages [19,20,21,22]. A major advantage of RT is that it can be targeted to the TME for local benefit, while limiting systemic effects. Additionally, local, intratumoral administration of Gal-3 inhibitors and a 4-1BB mAb agonist maximizes exposure of lymphocytes, microglia, and other immune cells within the TME [23,24,25]. This provided a strong preclinical rationale for combining an immune-priming dose of focal LDXR, locally delivered, non-cytotoxic doses of a Gal-3 inhibitor and an agonistic anti-4-1BB mAb to explore the effects of combining these three modalities on tumor response.

In this study, we evaluate this treatment combination in both in vitro (cell co-culture) and in vivo settings, using an orthotopic preclinical model of G3MB.

## 2. Materials and Methods

### 2.1. Human Tissue Samples

The Medical University of South Carolina (MUSC) Institutional Review Board (IRB) approved this study, allowing the acquisition of MB tissue samples from the Biorepository & Tissue Analysis Shared Resource, which is under the NCI-designated Hollings Cancer Center. Pediatric MB patients (mean age 8.8 ± 5.25 years) were selected based on clinical evaluation, magnetic resonance imaging (MRI), and pathological confirmation by a board-certified neuropathologist. A total of 15 MB tumor samples were obtained (5 each from Group 3, Group 4, and sonic hedgehog, SHH). Control postmortem normal cerebellar brain tissue blocks were obtained from the Harvard Brain Tissue Resource Center (Belmont, MA, USA). To the best of our knowledge, control patients were not taking any medication at the time of surgery.

### 2.2. Animals

C57BL/6J male mice (4–6 weeks old) were purchased from the Jackson Laboratory (Bar Harbor, ME, USA; #000664) for all phases of the study. The study was approved by the Institutional Animal Care and Use Committee (IACUC) at the Medical University of South Carolina.

### 2.3. Induction of Orthotopic Tumors and Treatment

MP1 MB cell lines, obtained from the Cheshire Laboratory (Stanford University, Stanford, CA, USA), are derived from Trp53-null cerebellar progenitor cells and are used to study aggressive, MYC-driven G3MB. Notably, the histological features of murine MP1 MB tumors resemble those of human large cell/anaplastic G3MB [26,27,28]. The cell line was cultured in NeuroCult™ proliferation medium (STEMCELL Technologies, Kent, WA, USA, #05761), along with human recombinant EGF (#78006.1), human recombinant bFGF (#78003.1), and heparin solution (#07980).

Mice underwent stereotactic trephination under anesthesia (ketamine/xylazine @ 80/10 mg/kg) using the following coordinates: 2 mm to the right and 2 mm posterior to lambda, below the cerebellar surface. Approximately 1 × 10^6^ mouse MP1 MB cells were suspended in 4–6 µL of media with Matrigel. The tumor cell mixture was slowly injected into the cerebellum at a steady rate over 30 s via a Hamilton syringe. The syringe was kept in place after completion of the injection for an additional two minutes before slow withdrawal. The burr-hole was sealed with bone wax, and the skin incision was approximated with staples, which were removed after 12 days. Control mice underwent the same surgical procedure but were implanted with Matrigel alone. Therapy began 2 weeks post-surgery.

For mice receiving radiation treatment, a single 1 Gy LDXR dose was delivered using a Varian TrueBeam linear accelerator (MUSC Radiation Oncology Facility) at 2 weeks post-transplantation. Alignment for radiation treatment was accomplished with an orthogonal laser system and a projected light field [29]. Regional or intratumoral delivery of low, non-toxic doses of TD139 [30] (200 µg/kg per day for 14 days) or anti-Gal-3 mAb (200 ng/kg per day for 14 days) with anti-4-1BB mAb (200 ng/kg per day for 14 days) was administered after LDXR via ALZET^®^ (Alzet LLC, Vacaville, CA, USA) osmotic pumps (84 µL at 0.25 µL/h) to overcome the blood-brain and blood-cerebrospinal fluid barriers, and to prevent mAb-related side effects and toxicity caused by tail vein intravenous administration. Animals were sacrificed 5–7 weeks after tumor cell implantation, and brain tissue was collected for biochemical and morphological analyses. Some animals in each group were included in a survival analysis. Western blot and immunohistochemistry (IHC) were performed to analyze changes in protein expression [29,30].

### 2.4. Cell Culture and Treatments

For our proof-of-concept studies, we developed methods to isolate distinct cell types from mouse brains, specifically focusing on primary neuronal isolation from normal brains and immune cell isolation (microglia and peripheral blood mononuclear cells, PBMCs) from tumor-bearing brains.

Mouse normal neurons were isolated from the cerebral cortex of embryonic (typically E15–E18) or neonatal (postnatal P0–P3) mice using the Miltenyi Biotech protocol. Mouse M2-microglia (10%), PBMCs (10%), MP1 (80%), or normal neurons (80%) were used in co-culture at the indicated ratios, based on the study goals, with ratios chosen to avoid toxicity to normal neurons. Isolation of microglia from adult tumor-bearing mouse brains involved fast, cold mechanical/enzymatic dissociation followed by Percoll gradient centrifugation (30%/70%) and optional magnetic sorting (CD11b+/CD45+) for high purity. The high myelin content necessitated thorough debris removal and density separation to separate microglia from MP1 cells and TAMs. Isolated cells retained microglial morphological (including a small cell body with branched processes) and functional characteristics. TAMs in the TME are often identified by CD11b/c, CD68, and Iba1 markers, showing a pro-tumoral (M2-like) phenotype. PBMC were isolated from tumor-bearing mice by collecting anticoagulant-treated blood (via heart puncture), followed by density gradient centrifugation (e.g., Ficoll-Paque 1.084) to separate mononuclear cells. Due to low volumes, blood from 3 to 5 mice was pooled. Red blood cells were removed using ammonium-chloride-potassium (ACK) lysis buffer, and remaining cells were washed with PBS. In TME models, microglia and blood often constituted a significant proportion (sometimes 10% or more) of the total tumor mass.

Cells were seeded and allowed to adhere for 24 h prior to LDXR, which was delivered using an X-RAD 320 (Precision X-ray) with 320-kV X-rays and a 2 mm aluminum filter, at a dose rate of 3.8 Gy/min (MUSC Radiation Oncology Central Facility). For immunomodulation, cells were exposed to a single 1 Gy dose. Analysis was performed 3 days after radiation exposure. For combination treatments, we observed the effects of immune priming with sublethal low-dose 1 Gy X-ray radiation, combined with an anti-Gal-3 mAb (200 nM) and an anti-4-1BB mAb (200 nM). Following 3 days of treatment of mouse M2-microglia with these combinations, media were collected, and levels of proinflammatory factors (cytokines and chemokines) were analyzed using a Proteome Profiler Mouse Cytokine Array Kit, Panel A (R&D Biosystems, Minneapolis, MN, USA, #ARY006) according to the manufacturer’s instructions. Treated PBMC cells were analyzed by Western blot. Co-culture experiments were performed in triplicate and repeated at least three times at different passage numbers. Cell death was assessed by TUNEL assay, as previously described [29,30,31].

For both in vivo and in vitro experiments, two different Gal-3 blocking strategies were used. It is important to note the differences between Gal-3 inhibition via glycomimetics, such as TD139, and antibody-mediated neutralization using anti-Gal-3 mAbs. While both strategies yield comparable anti-fibrotic and anti-inflammatory outcomes in preclinical models of disease—such as the attenuation of collagen deposition and macrophage recruitment—their pharmacokinetic profiles differ widely. TD139 binds competitively to the carbohydrate recognition domain of Gal-3 and is optimal for localized, high-concentration delivery, whereas the anti-Gal-3 mAb provides systemic neutralization and targets a broader array of Gal-3-mediated protein–protein interactions. Throughout this manuscript, ‘Gal-3 inhibitor’ and ‘Gal-3 blockade’ are used interchangeably to refer to Gal-3 targeting with TS139.

### 2.5. Immunohistochemistry

Cryosections were processed for antigen retrieval in citrate buffer (10 mM Sodium Citrate, 0.05% Tween 20, pH 6.0). Sections were washed in 0.01 M phosphate-buffered saline + 0.1% Triton-X-100 (PBST, pH 7.4) and blocked with normal serum for 30 min at room temperature. Terminal deoxynucleotidyl transferase (TdT)-mediated dUTP nick-end labeling (TUNEL) assay was performed using the Promega Dead End Fluorometric TUNEL kit (Promega Corp., Madison, WI, USA), according to the manufacturer’s instructions. Standard laboratory immunohistochemistry (IHC) techniques were used to identify histopathological characteristics of tumor samples [29,30,31]. Slides were mounted with a Vector Shield with DAPI nuclear stain. A minimum of three images (200× magnification) were captured for each section using an Olympus fluorescent microscope (Olympus America Inc, Center Valley, PA, USA). For IHC, primary rabbit monoclonal antibodies against Gal-3 (#EPR19244), CD3 (#ab135372), Egr2 (#ab232695), MYC (ab32072), NeuN (#ab177487), CD38 (#ab216343), GFAP (#ab68428), and CD8 (#ab237723) were purchased from AbCam (Cambridge, MA, USA); 4-1BB (#31-1363-00) and MAB328 (# MAB328-I) were purchased from RevMAb Biosciences, Burlingame, CA, USA and Sigma-Aldrich, St. Louis, MO, USA, respectively. A minimum of three 200× magnification images were captured for each section using a Zeiss fluorescent microscope (Carl Zeiss, Inc., White Plains, NY, USA) from the Storm Eye Institute, MUSC. All images were compiled and processed using Adobe Photoshop Elements.

### 2.6. Western Blot Analysis

Western blotting was performed using standard laboratory protocol [29,30,31]. Gels were stained with Ponceau S immediately after transfer to confirm equal protein loading and transfer efficiency across all lanes. To conserve reagents, molecular weight markers on the membrane were used to indicate where each protein of interest would be located and a horizontal band at that location was excised and incubated with the appropriate primary IgG antibody: β_2_-microglobulin (#MAB8325), active caspase-3 (#MAB835), CXCL-7 (#MAB1091), CXCL7 (#MAB2164), R&D Systems, Minneapolis, MN, USA; H-2Kb (MHC Class I) (Clone Y-3, #Y200-1MG), CD25 (Clone PC61.5, #14-0251-86), CXCL-6 (#245114), Thermo Fisher Scientific Inc., Waltham, MA, USA; Bcl-2 (C-2, #SC-7382), Myc (9E10, #sc-40), Gal-3 (B2C10, #sc-32790), MDR1/ABCB1(D-11, #sc-55510), GAPDH (G-9, #sc-365062) Santa Cruz, CA, USA; GRM8; MHC class II (clone M5/114, #MABF33, Sigma-Aldrich, MO, USA); CD69 (Clone 8B6, #NBP1-51607, Novus Biologicals, Centennial, CO, USA); or MRP-1 (#ab180960, Abcam, Cambridge, MA, USA); Antibody (YA3484, #HY-P83787) Med Chem Express, Monmouth Junction, NJ, USA. Anti-Glyceraldehyde-3-phosphate dehydrogenase GAPDH (G-9) was used to standardize protein loading.

Western blots were incubated with ECL detection reagents (Amersham Pharmacia, Buckinghamshire, UK) and visualized using an Alpha Innotech FluorChem FC2 Imager (Cell Biosciences, San Leandro, CA, USA). Immunoreactive bands were quantified using NIH ImageJ software (version 1.45). Antibody specificity was confirmed by the presence of a single band of the predicted size in Western blots and by the absence of signal in negative controls where the primary antibody was omitted. The membranes were not intentionally cut to exclude any bands, and no data were selectively removed. The original, unedited, uncropped blot strips are included in the manuscript; densitometry readings for each protein band are provided in a Supplemental Figure S1. Each experiment was completed five times to ensure data reliability, accuracy, and reproducibility.

### 2.7. Flow Cytometry

MP1 tumor-bearing mice with or without 14-day treatment were transcardially perfused. Brains were removed, and the hemispheres were dissociated in cold FACS buffer (1× PBS, 2% FBS, 1 mM EDTA) using a cell strainer and a mechanical homogenizer (e.g., Dounce or GentleMACS). Following mechanical and enzymatic dissociation of the brain, single-cell suspensions were collected and resuspended in 150 μL of ice-cold PBS containing 0.5% BSA per brain. To remove myelin debris, 30 μL of Myelin Removal Beads II (#130-096-733, Miltenyi Biotec, San Jose, CA, USA) were added, and the suspension was incubated for 15 min at 4 °C. Cells were then washed with 1 mL of buffer, centrifuged at 300× *g* for 10 min, and the supernatant was discarded. The cell pellet was resuspended in 500 μL of buffer and applied onto LS columns placed within a MACS magnetic separator (Miltenyi Biotec, CA, USA). The effluent, containing the unlabeled, myelin-depleted cell fraction, was collected. To maximize recovery, columns were washed twice with 3 mL of buffer. The final myelin-depleted suspension was centrifuged at 300 μg for 10 min at 4 °C. The pellet was washed with cold PBS and resuspended in FACS buffer for flow analysis. Cell viability was determined by MTT [3-(4,5-dimethylthiazol-2-yl)-2,5-diphenyltetrazolium bromide] assay (#475989, Sigma-Aldrich, St. Lois, MO, USA).

For flow cytometry analysis, the cell suspension was incubated with Fc Block (anti-CD16/32) (#14-0161-82, Thermo Fisher Scientific Inc., MA, USA) for 10 min to prevent nonspecific binding. Cells were then washed and stained with anti-CD8 (#100706), anti-CD4 (#100412), anti-CD38 (#102717; Biolegend, San Diego, CA, USA), or anti-Egr2 (#53-6691-82; Thermo Fisher Scientific, MA, USA) antibodies for 30 min at 4 °C in the dark. After washing, cells were resuspended in 300 μL staining buffer. Cells were sorted on a BD LSRFortessa™ flow cytometer (BD Biosciences, Franklin Lakes, NJ, USA). Compensation was calculated using single-stained compensation beads. Data were analyzed using FlowJo™ Software (version 11) (BD Biosciences).

### 2.8. Statistical Analysis

Statistical analyses were performed using StatView software Version 4.5 (Abacus Concepts, Inc., Berkeley, CA, USA). Analysis of Variance (ANOVA) followed by Fisher’s post hoc test was used to determine whether significant differences existed between groups. Significance is expressed as * *p* < 0.05/** *p* < 0.01.

## 3. Results

### 3.1. Gal-3 Is Highly Expressed in Human MB Tumor Cells, Microglia, and T-Cells

**Figure 1:**
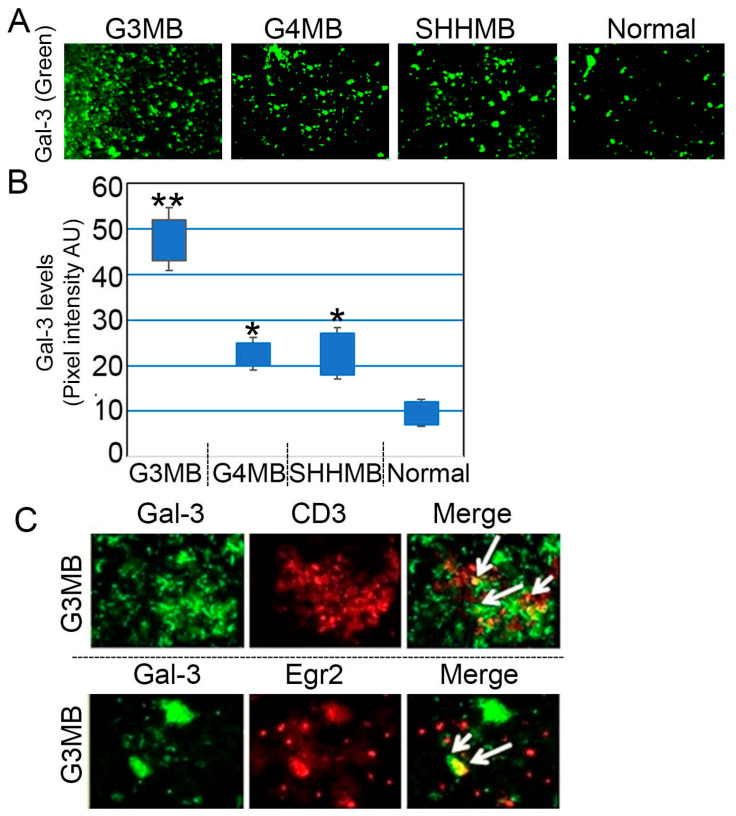
Gal-3 is upregulated in primary human pediatric medulloblastoma. (**A**) Immunofluorescence imaging data show higher levels of Gal-3 expression (green) in G3MB and G4MB tumor tissue compared to normal non-tumor tissue. There is a partial increase in Gal-3 expression in G4MB and SHHMB tissue. (**B**) Bar graph of Gal-3 levels in tissue. (**C**) Immunofluorescence imaging showing Gal-3 expressed (green) in Tumor-infiltrating CD3+T-cells or Egr2 (M2 phenotype) microglia (red) within G3MB tumors). Arrows show co-localization of Gal-3 expression with CD3+T-cells (upper panel) and Egr2 positive (M2 phenotype) microglia within G3MB tumor (bottom panel) (200× magnification). * *p* < 0.05/** *p* < 0.01 compared to normal. (n = 5).

To determine the differential expression of Gal-3, we compared staining intensities across MB subgroups. G3MB (MYC-amplified) tumors exhibited the highest Gal-3 expression among MB subtypes, with a statistically significantly higher intensity than SHH MB (*p* < 0.05) and G4MB (*p* < 0.05) counterparts (Figure 1A,B). Gal-3 expression was remarkably uniform in all seven G3MB cases, unlike the heterogeneous expression pattern observed in SHH tissues. In contrast, Gal-3 was much lower than in normal cerebellar tissues.

Immunostaining with microglia (Egr2) and T-cell (CD3) markers was used to identify the cell populations expressing Gal-3 within the G3MB tumor TME. We demonstrated that Gal-3 was expressed in M2-microglia, and that these microglia were present within the tumor core (Figure 1C). Furthermore, we observed that a distinct subset of tumor-infiltrating T-cells (CD3+) also expressed Gal-3 (Figure 1C). These findings suggest that Gal-3 is expressed by tumor cells, TAMs, and T cells within the G3MB core, revealing a distinct tumor-specific expression pattern not found in healthy brain tissue. The high Gal-3 expression in G3MB highlights its potential as a specific therapeutic target for this group.

### 3.2. LDXR Induces Immunomodulatory Phenotypes in the MP1 Microenvironment

**Figure 2:**
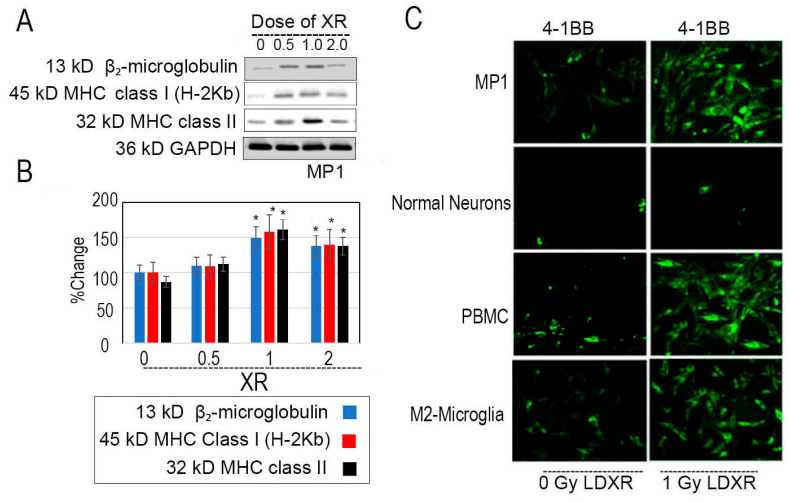
(**A**) Western blot analysis of the expression of β-2 microglobulin, MHC Class I (H-2Kb), and MHC class II at protein levels in MP1 cell line 72 h after X-ray radiation doses (0 Gy, 0.5 Gy, 1.0 Gy, or 2.0 Gy). GAPDH expression was measured to ensure that equal amounts of the sample were loaded in all lanes. (**B**) quantitative levels of expression of β-2 microglobulin, MHC Class I (H-2Kb), and MHC class II after LDXR. (**C**) Immunofluorescence imaging showed higher levels of 4-1BB expression (green) in MP1, mouse M2-Microglia, and mouse PBMCs in 1 Gy LDXR-treated cells compared to untreated cells. There was no change in 4-1BB expression in 1 Gy LDXR-treated mouse normal neuron cells compared to untreated cells. (200× magnification). * *p* < 0.05 compared to normal. (n = 5).

To investigate the immunomodulatory effects of radiotherapy, we used Western blot to analyze the expression of immunological markers following X-ray exposure at different doses. Our analysis demonstrated that low-dose (1Gy and 2Gy) radiation induced a significant (*p* < 0.05) upregulation of 13 kD β-2 microglobulin, 45 kD MHC Class I (H-2Kb), and 32 kD MHC Class II molecules on the surface of MP1 cells compared with untreated controls (Figure 2A,B). Furthermore, LDXR (1Gy) exposure promoted the expression of the co-stimulatory receptor 4-1BB (CD137) within the TME, specifically on TAMs and PBMCs, but not on neurons (Figure 2C). These findings indicate that LDXR not only enhances the antigen-presenting machinery of G3MB cells but also induces a pro-inflammatory profile in the surrounding immune microenvironment, creating a potential target for combined immune-radiation therapy.

### 3.3. LDXR with 4-1BB Activation and Gal-3 Blockade Reprograms the Tumor Microenvironment Toward an Antitumor Phenotype

**Figure 3:**
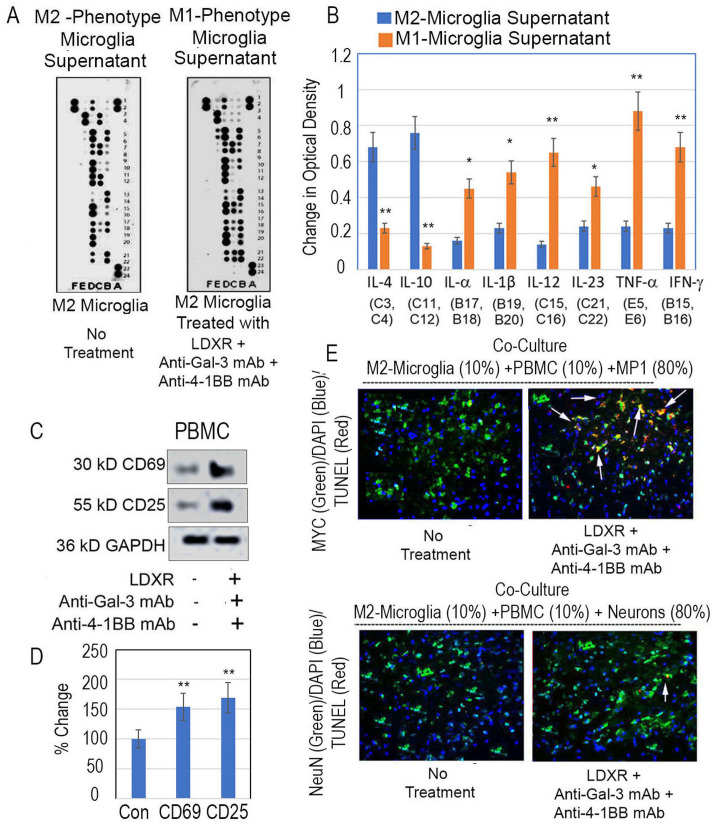
Low-dose radiation (1 Gy LDXR) + anti-4-1BB mAb (200 nM) + Gal-3 inhibitor (anti-mouse Gal-3 mAb:200 nM) enhanced antitumor immunity. (**A**) Proteome Profiler Mouse Cytokine Array spot showed that treatment of mouse M2-microglia caused conversion from M2 to M1 phenotype. (**B**) Quantitative densiometry expression data from cytokine spot array (average of three experiments with duplicate spots for each protein). Coordinates for each spot in (**A**) are provided under the chart. (**C**) Western blot analysis demonstrated that treatment induced CD69 and CD25 (IL-2Rα) expression in T cells, which are reliable indicators of T-cell activation. (**D**) Quantitative densitometry expression data from western blots. Con = control (**E**) Treatment induced MP1 cell death but did not affect normal neurons in co-culture models. Top panel: 80% MP1 + 10% PBMC + 10% M2-Microglia; histolabeling of DAPI (nuclei: blue) + MYC (MP1 marker: green) + TUNEL (red). Bottom panel: 80% Normal neurons + 10% PBMC + 10% M2-Microglia; histolabeling of DAPI (nuclei: blue) + NeuN (Neuron marker: green) + TUNEL (red). Arrow indicates TUNEL positive cells, an indicator of cell death * *p* < 0.05/** *p* < 0.01 compared to control (n = 5).

To elucidate the mechanism underlying the effects of the triple combination therapy (1 Gy LDXR + anti-Gal-3 mAb + anti-4-1BB mAb), we analyzed cytokine profiles in mouse microglia. Combination therapy significantly modulated the cytokine landscape, characterized by decreased levels (*p* < 0.01) of M2-macrophage-associated factors (IL-4 and IL-10) and increased expression of M1-macrophage-driven pro-inflammatory cytokines [IL-α (*p* < 0.05), IL-1β (*p* < 0.05), IL-12 (*p* < 0.01), IL-23 (*p* < 0.05), TNF-α (*p* < 0.01), and IFN-γ (*p* < 0.01)] (Figure 3A,B). Notably, the combined therapy promoted a shift from an immunosuppressive, M2-rich environment towards a high-functioning, M1-dominated phenotype, characterized by enhanced recruitment of activated lymphocytes. Consistent with these findings, reductions in tumor-associated inhibitory cytokines and an increased M1/M2 ratio (reflecting M1-to-M2 conversion) following the combined therapy correlated with enhanced therapeutic efficacy and a greater tumor reduction than with individual treatments. Furthermore, Western blot analysis confirmed that treatment of mouse PBMPBMCs with the combination therapy induced robust T-cell activation, as evidenced by significantly elevated expression of CD69 and CD25 (IL-2Rα), key activation markers (Figure 3C,D).

The combination therapy also induced significant cell death in MP1 medulloblastoma cells within the co-culture model. This effect was highly selective, as the triple therapy targeted the malignant population—comprising 80% of the model—while having no detectable impact on normal neuron cells (Figure 3E). Notably, this treatment efficacy was maintained in the presence of immune and stromal components, including microglia (10%) and PBMCs (10%), indicating that this robust antitumor response occurred within a complex microenvironment without apparent neurotoxicity.

### 3.4. Combination Therapy with 1 Gy LDXR, Gal-3 Inhibitor, and Anti-4-1BB Induces Robust MP1 Cell Death and Prolongs Survival

**Figure 4:**
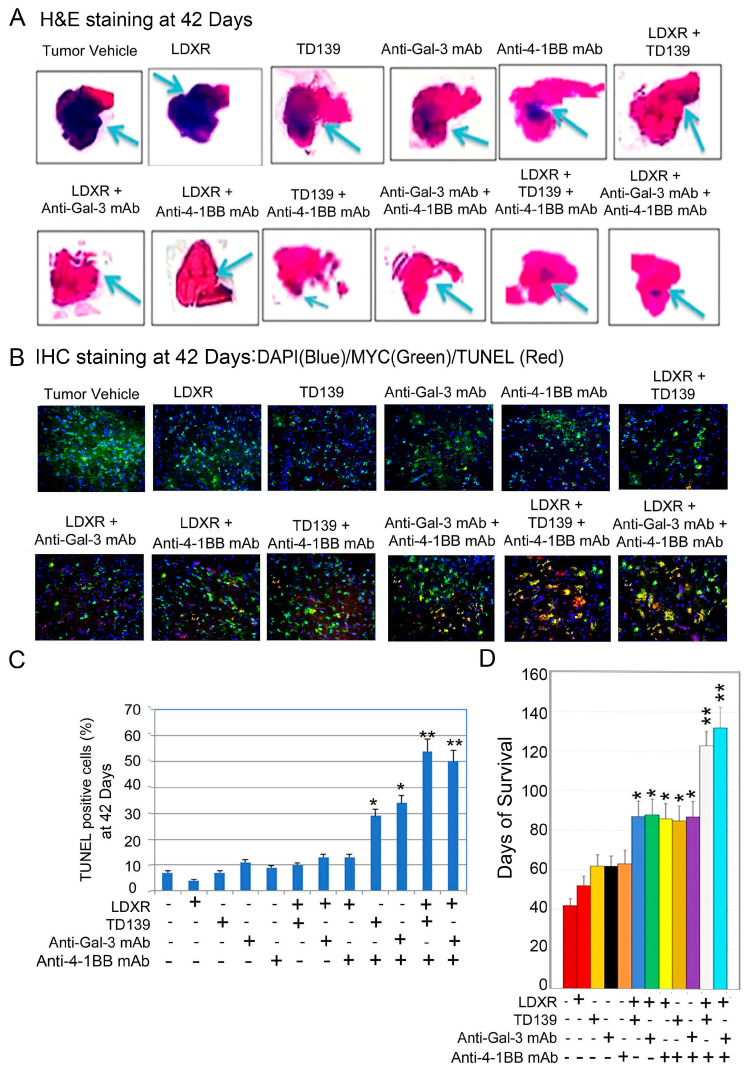
(**A**) Examination of H&E-stained tumor sections following 1 Gy LDXR + Gal-3 inhibitor (anti-mouse Gal-3 mAb:200 ng/kg per day for 14 days or TD139 200 µg/kg per day for 14 days) + anti-4-1BB mAb (200 ng/kg per day for 14 Days) treatment, 10× images. Tumor size was reduced by ~70% compared with untreated controls. Arrows and dark blue color indicate tumor location. (**B**) Histolabeling of DAPI (nuclei: blue) + MYC (Medulloblastoma cellular marker: Green/TUNEL (red). (**C**) Bar graph showing TUNEL positive cells (Yellow color in Right Panel) in the model treated with LDXR + Gal-3 inhibitor + anti-4-1BB mAb were significantly higher than with no treatment or a single treatment. (**D**) Survival of tumor-bearing mice treated with LDXR + Gal-3 inhibitor + anti-4-1BB mAb was significantly higher than in untreated mice. * *p* < 0.05/** *p* < 0.01 compared to untreated tumor vehicle (n = 5).

To evaluate the therapeutic efficacy of the triple combination [1 Gy LDXR) + Gal-3 inhibition (anti-mouse Gal-3 mAb or Gal-3 inhibitor (Gal3i) TD139) + immune checkpoint activation (anti-4-1BB mAb)] in the aggressive MP1 allograft MB model, we performed H&E staining, TUNEL assays, and comprehensive survival analysis. Following the 14-day treatment regimen, H&E-stained tumor sections revealed a marked reduction in tumor volume in the triple combination group compared with the control (untreated), single-agent, or double combination groups (Figure 4A). Histological analysis after the triple combination treatment showed profound morphological changes indicative of cell death, including nuclear condensation, reduced tumor cellularity, and increased necrotic areas within the tumor parenchyma. The enhanced anti-tumor activity was further validated by the TUNEL assay, which showed a significantly higher number of apoptotic cells in the tumor tissues of the triple-combination group (*p* < 0.05) compared to all other groups, suggesting that targeting Gal-3 with the immune checkpoint in conjunction with radiotherapy dramatically enhanced tumor cell apoptosis (Figure 4B,C). Furthermore, survival analysis demonstrated that mice treated with the triple combination had a significantly longer median survival time than those treated with LDXR (1Gy) + 4-1BB or Gal-3i (Figure 4D). These results indicate that the triple treatment acts in combination to reduce tumor burden and induce robust cell death, likely by overcoming Gal-3-mediated immunosuppression and activating T-cell-mediated responses in the MP1 MB microenvironment, resulting in prolonged survival.

### 3.5. Triple Combination Therapy Promotes Anti-Tumor Immunity and Spares Normal Brain Parenchyma

**Figure 5:**
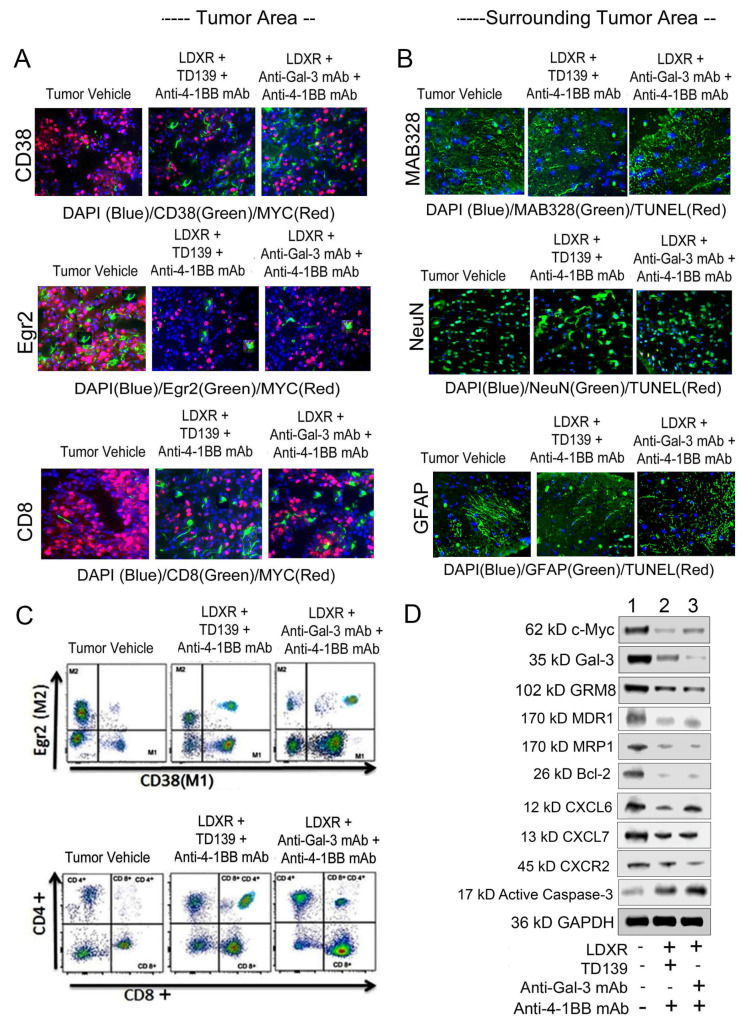
1 Gy LDXR + Gal-3 inhibitor (anti-mouse Gal-3 mAb:200 ng/kg per day for 14 days or TD139 200 µg/kg per day for 14 days) + anti-4-1BB mAb (200 ng/kg per day for 14 Days) treatments induced MB cell death via conversion of M2 TAMs to M1 phenotype. An increased number of CD8 T cells were observed within the tumor in MP1 allografted animal model with no effect on surrounding normal oligodendrocytes (MAB328), neurons (NeuN) and astrocytes (GFAP). (**A**) Histolabeling of DAPI (nuclei: blue) + CD38 (M1 macrophages marker: Green)/or Egr2 (M2 macrophages marker: Green) or CD8 (Cytotoxic Cell marker: Green) or MYC (Medulloblastoma cellular marker: red). (**B**) Histolabeling of DAPI (nuclei: blue) + MAB328 (Oligodendrocyte cellular marker: Green)/or NeuN (Neuron cellular marker: Green)/or GFAP (Astrocyte cellular marker: Green)/or /TUNEL (red). Yellow showed co-localization with cell death. (**C**) Representative flow cytometry dot plots show that the percentage of M1 phenotype microglia (CD38), CD8+, and CD4+ increased after treatment of the MP1 tumor. (**D**) Treatment with either LDXR + anti-Gal-3 mAb + anti-4-1BB mAb or LDXR + TD139 + anti-4-1BB mAb suppressed expression of c-Myc, Gal-3, GRM8, MDR1, MPR1, Bcl-2, CXCL6, CXCL7, and CXCR2. Increased expression of active caspase-3 was also observed in the MP1 allograft MB model. Quantitative densitometry data are provided in Figure S1. (n = 5) (200× magnification).

To further evaluate the triple combination therapy, we analyzed changes in the immune microenvironment in the MP1 allograft animal model after treatment. Immunohistochemical staining of tumor sections revealed a significant conversion of tumor-associated macrophages (TAMs) from an M2 (Egr2+) pro-tumoral phenotype to an M1 (CD38+) anti-tumoral phenotype (Figure 5A). Concurrently, quantification of tumor-infiltrating lymphocytes revealed a significant increase in CD8 T cell density within the tumor mass compared to controls (Figure 5A). Notably, high-resolution IHC analysis of the surrounding brain parenchyma showed no significant alteration in the expression of markers for oligodendrocytes (MAB328), neurons (NeuN), or astrocytes (GFAP) in the treated group compared to sham-treated animals (Figure 5B). Furthermore, flow cytometric analysis of dissociated MP1 brain tumors revealed that the treatment stimulated a robust immune response within the tumor microenvironment. Compared to controls, treated MP1 tumors exhibited a significantly increased percentage of M1-polarized microglia (defined as CD38+). Furthermore, infiltration of adaptive immune cells was increased, with higher percentages of CD8+ T cells in the treated group (Figure 5C). These data indicate that the treatment promoted a shift towards a pro-inflammatory microenvironment in MP1 brain tumors. Together, these results demonstrate that our triple combination treatment induced robust immunogenic death of G3MB cells through reprogramming of the TME, while sparing surrounding normal brain tissue.

To investigate the molecular mechanisms underlying the efficacy of the combined therapy, we performed Western blot analysis on tumor tissues from the MP1 allograft medulloblastoma (MB) model. Combination treatment significantly suppressed the expression of key regulatory proteins across several oncogenic and survival pathways. Specifically, we observed a marked reduction in the expression of the oncogenic driver c-Myc (62 kD) and the target protein Gal-3 (35 kD). This was accompanied by robust downregulation of multidrug resistance transporters MDR1 (170 kD) and MRP1 (170 kD), as well as the anti-apoptotic regulator Bcl-2 (26 kD), suggesting sensitization of the tumor to therapy (Figure 5D). Furthermore, the treatment effectively inhibited expression of chemokine signaling components, including CXCL6 (12 kD), CXCL7 (13 kD), and their receptor, CXCR2 (45 kD), as well as the metabotropic glutamate receptor GRM8 (102 kD) (Figure 5D). Consistent with the observed suppression of survival markers, combination therapy led to a significant increase in active caspase-3 (17 kD) expression (Figure 5D), indicating induction of apoptosis in the MP1 allograft MB model.

## 4. Discussion

Medulloblastomas are commonly considered immunologically cold and refractory to immunotherapy due to limited immune infiltration and antigen presentation. Impaired antigen presentation allows tumor cells to escape cytotoxic T cells. In this study, we demonstrate the importance of Gal-3 inhibition and the activation of M2 microglia and CD8+ T cells in controlling G3MB growth, and show that LDXR, which has previously shown limited success as a monotherapy for numerous cancers, acts as an adjuvant for TME-centered immunotherapy.

We initially used LDXR to induce MHC class I/II expression in tumor cells. The observed upregulation of MHC Class I and II, coupled with induced 4-1BB expression, suggests a reversal of common tumor-mediated immune-evasion strategies. Increased MHC-I/II density likely enhances recognition by CD8+ and CD4+ T cells, while 4-1BB acts as a critical costimulatory ‘second signal’ to sustain the effector phase. These data strongly suggest that 1 Gy LDXR shifted the tumor microenvironment toward a more highly immunogenic state. The combination of 1 Gy LDXR with an anti-4-1BB mAb and a Gal-3 inhibitor effectively triggered MP1 cell death while sparing normal neurons, indicating a highly selective and robust immune-mediated response. Taken together, these data demonstrate that our triple-therapy approach (LDXR + Gal-3 inhibition + 4-1BB activation) is a useful strategy for enhancing tumor control and immune activation, especially in immunologically “cold” or resistant cancers.

Building on this insight, we pursued a more translatable treatment approach, using ALZET osmotic pumps, which are frequently used to facilitate convection-enhanced delivery (CED) in preclinical research, to administer Gal-3 inhibitors and anti-4-1BB mAbs directly into brain tumors. CED remains an experimental technique, but recent clinical trials have demonstrated its promise for delivering therapeutic agents to patients with brain cancer [32]. Here, we show that CED of Gal-3 inhibitors and anti-4-1BB mAbs, combined with 1 Gy LDXR, significantly suppressed medulloblastoma tumor growth in the MP1 allograft model and improved overall survival. This therapeutic benefit is likely driven by a fundamental shift in the tumor immune landscape: the treatment effectively converts immunosuppressive M2 microglia into a pro-inflammatory M1 phenotype, which, in turn, activates intratumoral T cells. Critically, this potent anti-tumor effect was achieved with high selectivity, as we observed no detrimental effects on surrounding healthy neurons or oligodendrocytes. These findings suggest that this triple target approach can overcome the immunosuppressive TME in MB and effectively sensitize immunologically cold tumors to CD8+ T cell-mediated killing, while maintaining a favorable safety profile for neural tissue. These findings also validate CED as a clinically relevant therapeutic delivery method.

However, while our proposed mechanism highlights the dynamic roles of microglia and TAMs, it is critical to acknowledge the limitations of myeloid cell plasticity. Emerging evidence indicates that these cells do not undergo unrestricted phenotypic switching. Instead, their functional plasticity is constrained by continuous signals from the tumor microenvironment, distinct developmental origins (ontogeny), and entrenched epigenetic programs. Consequently, therapeutic strategies aimed at reprogramming these cells must account for these rigid physiological boundaries rather than assuming fluid transitions between activation states. We also acknowledge that the absence of functional T-cell assays is a further limitation. While our data demonstrate upregulation of the activation markers CD69 and CD25, these phenotypic markers do not directly confirm the functional status, proliferative capacity, or cytokine-secreting capacity of T cells. Future studies incorporating functional assays—such as intracellular cytokine staining, immunophenotyping (flow cytometry/RNA-seq), ELISPOT, and cytotoxicity assays—genetic ablation models, and multi-omics profiling will be necessary to comprehensively characterize the functional profiles of these activated T cells and macrophages in vivo. While our data demonstrates a profound phenotypic shift in CD8^+^ T cells and macrophages, an additional limitation of this study is the lack of functional depletion experiments. Future studies utilizing cell-specific depletion or knockout models will be required to define the precise mechanistic necessity of these individual populations. At the same time, future studies will need to address potential challenges, including drug resistance and dose-related toxicities, to fully harness the therapeutic potential of this novel therapeutic strategy.

The triple therapy significantly attenuated markers associated with drug resistance and the tumor microenvironment. Specifically, the reduction in MDR1, MPR1, and GRM8 expression suggests that inhibiting Gal-3 and activating 4-1BB might sensitize MB cells to radiation by bypassing standard resistance mechanisms. Furthermore, the suppression of pro-inflammatory and chemotactic factors, such as CXCL6, CXCL7, and CXCR2, may indicate remodeling of the TME toward a less tumor-supportive state. Coupled with elevated active caspase-3, these changes underscore a shift from tumor-promoting signaling to an environment favoring tumor cell death.

## 5. Conclusions

In summary, our data highlight the therapeutic potential of a triple-therapy strategy to enhance blockade of tumor growth, reprogram the TME to favor M1 macrophage polarization, and generate potent effector CD8+ T cell responses. With a better understanding of immune activation following LDXR, we can design rational clinical trials to maximize the combined effects of LDXR, anti-4-1BB agonists, and Gal-3 inhibitor treatments, thereby fostering a more robust anti-tumor immune response and improving outcomes in G3MB.

## Data Availability

The original contributions presented in this study are included in the article. Further inquiries can be directed to the corresponding author.

## Supplementary Materials

The following supporting information can be downloaded at: https://www.mdpi.com/article/10.3390/cancers18121890/s1, Figure S1: Treatments with either LDXR + anti-Gal-3 mAb + anti-4-1BB mAb or LDXR + TD139 + anti-4-1BB mAb showed (Quantitative bar diagram) suppressed protein expression (c-Myc, Gal-3, GRM8, MDR1, MPR1, Bcl-2, CXCL6, CXCL7, and CXCR2). There was also increased expression of active caspase-3 in the MP1 allograft MB model. ** *p* < 0.01 compared to untreated tumor vehicle (n = 7).

## Abbreviations

The following abbreviations are used in this manuscript:

ACK	ammonium-chloride-potassium
ANOVA	analysis of variance
APC	antigen presenting cell
G3MB	group 3 medulloblastoma
G4MB	group 4 medulloblastoma
Gal-3	galectin-3
Gal-3i	galectin-3 inhibitor
ECL	enhanced chemiluminescence
EGF	epidermal growth factor
H&E	hematoxylin and eosin
IACUC	Institutional Animal Care and Use Committee
IHC	Immunohistochemistry
IRB	Institutional Review Board
LDXR	Low-dose X-ray radiation
mAb	monoclonal antibody
MB	medulloblastoma
MDSC	myeloid-derived suppressor cell
MHC	major histocompatibility complex
MUSC	Medical University of South Carolina
MP1	mouse cerebellar tumor cell line
MRI	magnetic resonance imaging
MTT	3-(4,5-dimethylthiazol-2-yl)-2,5-diphenyltetrazolium
NCI	National Cancer Institute
PBMC	peripheral blood mononuclear cells
PBS	phosphate-buffered saline
RT	radiation therapy
SHH	sonic hedgehog (medulloblastoma type)
TAM	tumor-associated macrophages/microglia
TCR	T-cell receptor
TME	tumor microenvironment
TUNEL	terminal deoxynucleotidyl transferase dUTP nick end labeling
