# Supplementary figures and images for: Effects of Combining Immune-Priming Sub-Lethal Low-Dose Radiation with 4-1BB Activation and Gal-3 Blockade in In Vitro and Preclinical Group-3 Medulloblastoma Models
Source: Cancers (Basel). 2026 Jun 10;18(12):1890. doi: 10.3390/cancers18121890 (PMC13297374; doi:10.3390/cancers18121890)

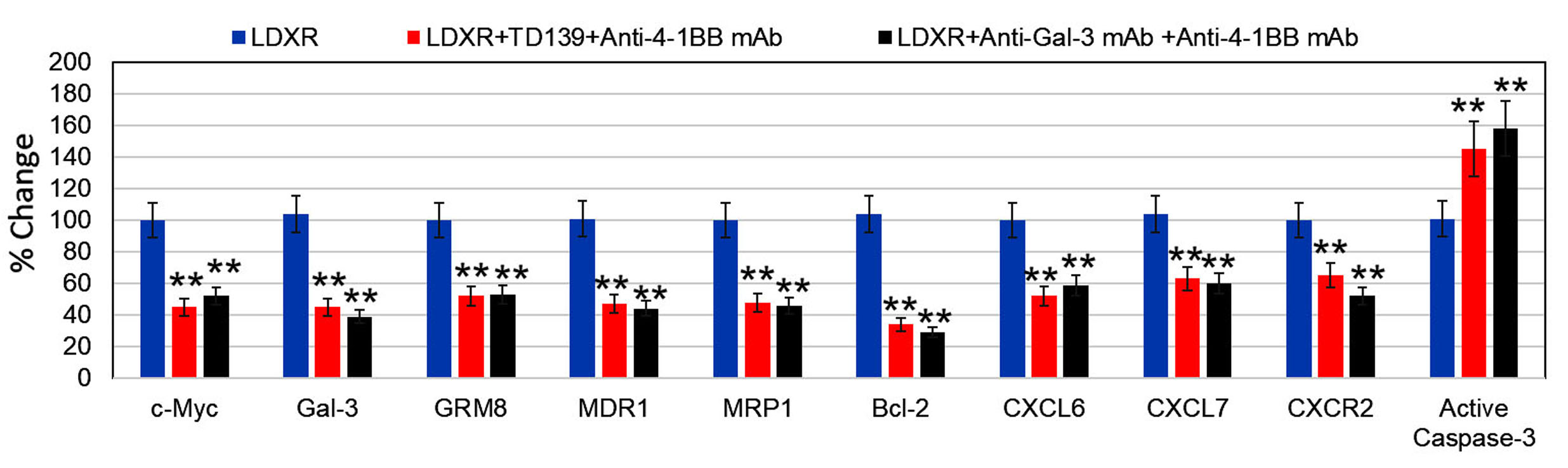

Supplement: Supplementary file 1 [file cancers-18-01890-s001.zip › cancers-4344430-supplementary.jpg]
